# Spatial distribution and habitat selection of *culicoides imicola*: The potential vector of bluetongue virus in Tunisia

**DOI:** 10.4102/ojvr.v88i1.1861

**Published:** 2021-08-16

**Authors:** Ben H. Thameur, Sghaier Soufiène, Heni Haj Ammar, Salah Hammami

**Affiliations:** 1 Ministry of Agriculture of Tunisia, General Directorate of Veterinary Services, CRDA Nabeul, Tunisia; 2 Tunisian Veterinary Research Institute, R Djebel El Akhdhar Errabta, Tunisia; 3 Ministry of Agriculture of Tunisia, General Directorate of Veterinary Services, Rue Alain Savary, Tunisia; 4 Ecole Nationale de Médecine Vétérinaire de Sidi Thabet, IRESA, University of Manouba, Sidi Thabet, Tunisia

**Keywords:** Tunisia, bluetongue virus, *C. imicola*, ENFA, ecological-niche factor analysis, MADIFA, Mahalanobis distances factor analysis, ecological niche model, maxEnt

## Abstract

The increasing threat of vector-borne diseases (VBDs) represents a great challenge to those who manage public and animal health. Determining the spatial distribution of arthropod vector species is an essential step in studying the risk of transmission of a vector-borne pathogen (VBP) and in estimating risk levels of VBD. Risk maps allow better targeting surveillance and help in designing control measures. We aimed to study the geographical distribution of *Culicoides imicola*, the main competent vector of Bluetongue virus (BTV) in sheep in Tunisia. Fifty-three records covering the whole distribution range of *C.imicola* in Tunisia were obtained during a 2-year field entomological survey (August 2017 – January 2018 and August 2018 – January 2019). The ecological niche of *C. imicola* is described using ecological-niche factor analysis (ENFA) and Mahalanobis distances factor analysis (MADIFA). An environmental suitability map (ESM) was developed by MaxEnt software to map the optimal habitat under the current climate background. The MaxEnt model was highly accurate with a statistically significant area under curve (AUC) value of 0.941. The location of the potential distribution of *C. imicola* is predicted in specified regions of Tunisia. Our findings can be applied in various ways such as surveillance and control program of BTV in Tunisia.

## Introduction

The spread of disease vectors and the growing number of vector-borne diseases (VBDs) pose a major threat to human and animal health. Despite considerable success in controlling communicable diseases, VBDs have caused and still cause damage on different continents. Bluetongue (BT), an emerging and re-emerging VBD, is caused by double-stranded ribonucleic acid (RNA) orbiviruses of the Reoviridae family. Bluetongue virus (BTV) infects domestic and wild ruminants and it is transmitted by biting midges of the genus *Culicoides* (Diptera: Ceratopogonidae). Between 1999 and 2002, BTV serotype 2 epizootics have occurred in Tunisia (Ben Fredj et al. 2003; Hammami 2004). In 2006 and 2009, Tunisia’s General Directorate of Veterinary Services (DGSV) warned of incursion of two additional serotypes: BTV1 and BTV4, successively (Lorusso et al. 2013; Sghaier et al. 2014). In 2016, a novel BT serotype 3 was reported (Lorusso et al. 2018). Genome sequencing revealed two unrelated western strains of BTV-3, one circulating in Cap-Bon (Northwest of Tunisia) and neighbouring areas, and the other circulating nearby the border with Libya. *Culicoides imicola* is the main potential vector of BT in Tunisia (Hammami 2004; Sghaier et al. 2014).

Determining the spatial distribution of arthropod vector species is an essential step in studying the risk of transmission of a vector-borne pathogen (VBP) and in estimating the level of risk. Geospatial technologies are commonly used to evaluate vector patterns or human or animal case distributions and to estimate the risk of disease transmission based on entomological, epidemiological and environmental factors. Diverse modelling methods have been developed and are commonly used to predict the geographic range of a species given presence-only (PO) occurrence data and environmental variables assumed to influence its distribution (Baasch et al. 2010; Bateman, VanDerWal & Johnson 2011; Güthlin et al. 2011).

In our study, we used two complementary protocol analyses: Ecological Niche Factor Analysis (ENFA; Hirzel et al. 2002) and Mahalanobis Distances Factor Analysis (MADIFA; Calenge & Basille 2008), to explore the relationship between the ecological niche and the availability of habitats of *C. imicola*. After selecting the variables associated with the ecological niche of *C. imicola*, we used the Maximum Entropy (MaxEnt) approach to model *C. imicola* presence and mapping habitat suitability (HS) (Phillips, Anderson & Schapire 2006). We expect this approach to prove to be an effective tool to assess and visualise the risk of the establishment and the spread of BT in Tunisia.

## Materials and methods

### Presence points

A large entomological monitoring was carried out over the entire territory of Tunisia between 2017 and 2019 to describe the diversity, distribution and seasonal dynamics of *Culicoides* in relation with BT cases. Seventy-two sites were monitored during two periods; 34 sites from August 2017 to January 2018 and 38 sites from August 2018 to January 2019. Two-night catches per site were performed monthly using black ultraviolet (UV)-light traps manufactured by the Onderstepoort Veterinary Institute (OVI, South Africa). Insects were collected in a beaker containing 200 millilitres (mL) – 250 mL of water and 3–4 drops of detergent, as a wetting agent. Each catch was transported to the laboratory, then covered and preserved in 70% ethanol for further study. *Culicoides imicola* were identified according to wing patterns, using stereoscopic microscope and taxonomic keys and subsequently confirmed by mounting specimens on microscope slides (Delécolle 1985). *Culicoides. imicola* is identified in 53 sites (Presence points, white dots in Figure 6).

### Bioclimatic variable

To determine which environmental variables most influence the distribution of *C. imicola*, the main vector of BT in Tunisia, we included 12 bioclimatic variables in our model (Table 1). The selection of environmental and climatic predictors used in the analysis was based on the literature review and association already proved with the disease (Calvete et al. 2009; Ciss et al. 2019; Van Doninck et al. 2014). The bioclimatic data, with a spatial resolution of 30 arc seconds (~1 kilometres [km]), were downloaded from the World Climate website (http://www.worldclim.org/current) and averaged over a 50 year period between 1950 and 2000 at the same spatial resolution. First WorldClim data files are converted to Esri ASCII format in DIVA-GIS (http://www.diva-gis.org) and trimmed in the region of Tunisia. The final step was to convert the trimmed grid files to the ESRI ASCII format. The files are then ready to be used in R-Studio and in MaxEnt software.

**TABLE 1:** Bioclimatic variables used in the model.

Bioclimatic variable	Significance
BIO3	Isothermality (BIO2/BIO7) (* 100)
BIO4	Temperature seasonality (standard deviation *100)
BIO5	Max temperature of warmest month
BIO6	Min temperature of coldest month
BIO8	Mean temperature of wettest quarter
BIO10	Mean temperature of warmest quarter
BIO12	Annual precipitation
BIO13	Precipitation of wettest month
BIO14	Precipitation of driest month
BIO15	Precipitation seasonality (coefficient of variation)
BIO16	Precipitation of wettest quarter
BIO18	Precipitation of warmest quarter

### Modelling habitat suitability

We used two complementary model analyses; ENFA and MADIFA, to explore the relationship between the ecological niche of *C. imicola* and the availability of habitats (Calenge & Basille 2008). The ENFA and MADIFA multivariate analyses were performed using the functions ‘enfa’ and ‘madifa’ of the package adehabitatHS (Calenge 2006) available in the open-source software R (R Development Core Team 2009).

The ENFA analyses the data in relation to the environment, that is, what is available for the species; the MADIFA focuses on the habitat, that is, what the species uses. In ENFA, the components have direct ecological meaning. The first factor is the marginality: the direction in which the species niche differs at most from the available conditions in the global area. The higher the absolute value of marginality, the more the species habitat differs from the study area. A positive marginality means that the species prefers higher-than-mean values on the ecological variable. Specialisation factors indicate how restricted the species’ niche is in relation to the study area. The MADIFA, in contrast to the ENFA, locates the directions in ecological space for which the niche is narrowest relative to the available environment (Calenge & Basille 2008). It combines species marginality and specialisation into a unique measure of HS. The two methods make it possible to predict the potential habitat for the species and to build environmental suitability maps (ESM) (Basille et al. 2008).

After selecting environmental predictors associated with the ecological niche of *C. imicola*, we used the MaxEnt approach to model *C. imicola* presence and mapping ESM.

### Maximum entropy model: Mapping of the optimal regions for *Culicoides imicola*

In this study, we used the maximum entropy model (MaxEnt version 3.4.1 [Phillip et al. 2006]; http://www.cs.princeton.edu/wschapire/MaxEnt/) because it has been shown to perform better with small sample sizes compared with other modelling methods (Elith et al. 2006; Kumar & Stohlgren 2009; Pearson et al. 2007). Maximum Entropy uses PO data to predict the distribution of a species based on the theory of maximum entropy. The programme attempts to estimate a probability distribution of species occurrence that is closest to uniform whilst still subject to environmental constraints (Elith et al. 2011). Maximum Entropy takes a list of species presence locations as input, often called PO data, as well as a set of environmental predictors across a user-defined landscape that is divided into grid cells. From this landscape, MaxEnt extracts a sample of background locations that it contrasts against the presence locations. Presence is unknown at back-ground locations. The relative occurrence rate (ROR) is the relative probability that a cell is contained in a collection of presence samples. The ROR corresponds to Maxent’s raw output. Maximum Entropy can be used to predict the probability of presence only by using a transformation of the ROR, called logistic output (Phillips & Dudik 2008). Maximum Entropy predicts RORs as a function of the environmental predictors at that location. These RORs sum to unity across the landscape because the denominator is a sum of the RORs over all grid cells in the study (called normalisation). Normalisation ensures that the occurrence rates are in fact RORs.

To reduce multi-collinearity amongst the bioclimatic variables used in Maxent, highly correlated variables (*r* ≥ 0.85 Pearson correlation coefficient) were eliminated from further models (Graham 2003). Jackknife analyses were performed to determine variables which, when omitted, reduce the model reliability. We used the area under the receiving operator curve (AUC) to evaluate model performance. The value of AUC ranges from 0 to 1. An AUC value of 0.50 indicates that the model did not perform better than random, whereas a value of 1.0 indicates perfect discrimination (Swets 1988). The model with the highest AUC value was considered the best performer.

## Results

### Environmental factors associated with the collection data

**FIGURE 1:**
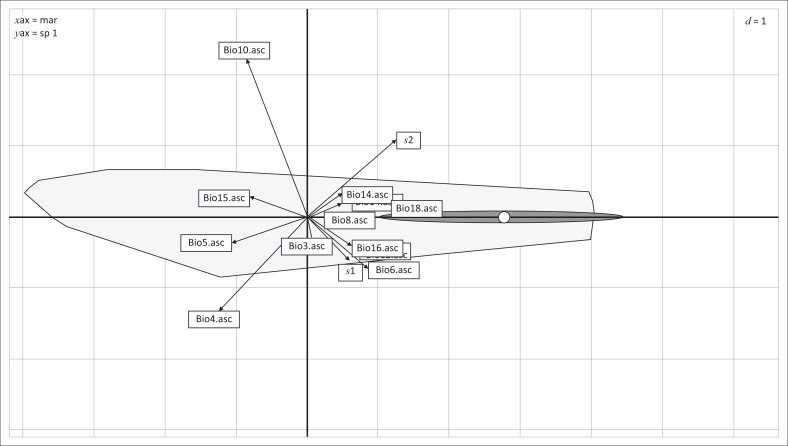
Results of the ecological-niche factor analysis for *C. imicola* in Tunisia.

As illustrated by the ENFA (Figure 1) the centroid of the ecological niche was far from the centroid of the available habitat, meaning that used and available distributions differed with respect to their mean (marginality, first axis) and variance (specialisation, second axis). The ENFA method provides for our presence data an overall marginality of *m* = 7.706, showing that *C. imicola* habitat is very different from the mean habitat of the study area (*C. imicola* presence needs specific ecological conditions) and an overall specialisation of *s* = 2.663, which means that we found *C. imicola* in a small range of conditions. If we observe the marginality coefficients of every eco-geographical variable (Table 2), Bio4, Bio18, Bio5, Bio6 and Bio10 correlate with the marginality axis at 39%, 38%, 32%, 27% and 26%, respectively. Positive values mean that the values of variables inside site areas are greater than values in the whole study area. Bio 10 correlates with the first specialisation axis (71%, Table 2, Figure 1). Bio4 and Bio6 also correlate with this axis but to a lesser extent showing 42% and 23%, respectively. The values of specialisation factors indicate that *C. imicola* sites have a small range of Bio10, Bio4 and Bio6 (Table 2).

**TABLE 2:** Correlations (%) between variables and axes for the ecological niche factor analysis (marginality and 1st axe of specialisation) and the Mahalanobis distances factor analysis (1st and 2nd axes).

ENFA Mar ENFA Spe1	mad_Axis1 mad_Axis2
bio3 0.02070506 −0.09406696	−0.06502169 −0.12137425
bio4 −0.39798036 −0.42615219	−0.09662020 −0.30769982
bio5 −0.32592704 −0.10882675	0.31462231 0.19317539
bio6 0.27896568 −0.23735255	0.18795900 −0.01812637
bio8 0.04556967 −0.02382029	−0.02006778 −0.02573204
bio10 −0.26992329 0.71431994	−0.45223501 0.35308768
bio12 0.24258736 −0.16201645	−0.52172312 −0.51742373
bio13 0.20677530 0.07731757	0.17716050 −0.02436514
bio14 0.16167924 0.09887452	−0.05398270 0.03263953
bio15 −0.23187646 0.09064017	−0.36316646 −0.20323736
bio16 0.20656889 −0.13003976	0.44834361 0.64450732
bio18 0.38858103 0.02718194	−0.08834506 −0.03910680

ENFA, ecological-niche factor analysis.

The ecological-niche factor analysis results displayed by the marginality axis (*x*-axis) and first specialisation axis (*y*-axis). Marginality was strong as the dot representing the centroid of the distribution of used units shifted from the origin of the axes, the centroid of the distribution of available weights. The polygons correspond to the minimum convex polygons of the distributions of available (light grey area) and used (dark grey area) resource units. The polygon of the used habitat (realised niche) was narrow in its extent on the *y*-axis meaning that specialisation was also high. The direction and length of the arrows are a metric of the contribution of the variables to marginality and specialisation.

The results of MADIFA identified additional significant ecological variables in the study area, as it identified the directions of the ecological space where Mahalanobis distances were the largest, corresponding to the environmental conditions scarcely used by *C. imicola.*

**FIGURE 2:**
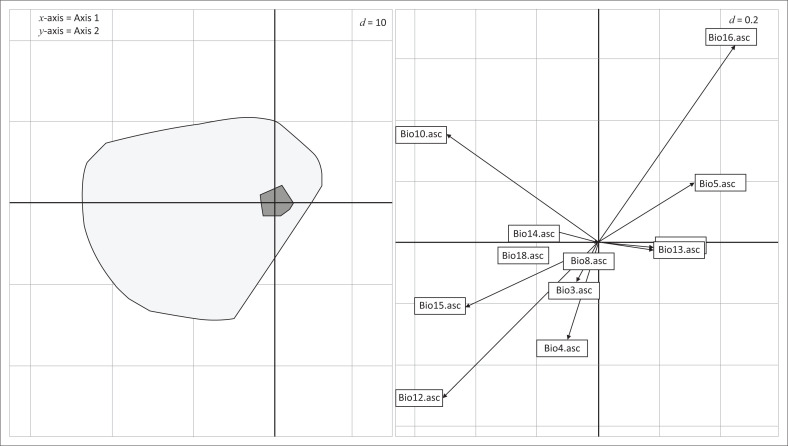
Scatter niche diagram of the cloud of available (grey circle) and used (black squares) points on the first two axes of the Mahalanobis distances factor analysis. The black points correspond to the ecological variables used by the *C. imicola*. Grid lines (separated by a distance of 0.2) can be used to measure the correlations between ecogeographical variables and significant factors (i.e. abscissa and ordinate axes) on the graph for each analysis.

As illustrated by the scatter niche of MADIFA (Figure 2), the used points are distributed in the restricted area of the factorial axes found by the analysis. This confirms that through analysis an interesting direction could be identified. The black dots (used habitats) are significantly offset on the first and second axis from the centre of the grey area and occupy part of the middle right sector of the available habitat. The first axis (abscissa) of MADIFA was strongly correlated with Bio12, Bio10 and Bio16 and the second axis (ordinate) was strongly correlated with Bio16, Bio12 and Bio10 (Table 2, Figure 2). These variables affect the position of the availability niche in relation to the used niche for the first and second factors, respectively.

**FIGURE 3:**
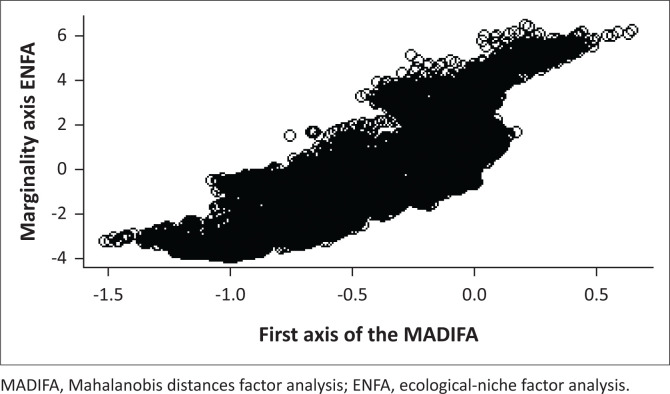
Correlation between the first axis of the Mahalanobis distances factor analysis and the marginality axis of the ecological niche factor analysis.

The results of ENFA and MADIFA are complementary as the marginality axis of the ENFA and the first component of the MADIFA were significantly correlated (rho = 0.689) (Figure 3).

During this procedure, variables that did not contribute to the ecological niche or highly correlated variables (with a coefficient of correlation higher than 0.95), were not used in the statistical analyses in MaxEnt.

### Maximum entropy model: Mapping of the optimal regions for *Culicoides imicola*

The reduction of predictor variables resulted in the inclusion of only height variables for MaxEnt model. Table 3 gives estimates of relative contributions of the environmental variables to the MaxEnt model.

**TABLE 3:** Relative contributions of the environmental variables to the Maximum Entropy model.

Variables bioclimatiques	Signification	Percent contribution
bio18	Precipitation of warmest quarter	61
bio10	Mean temperature of warmest quarter	14.5
bio12	Annual precipitation	10.6
bio6	Minimum temperature of coldest month	4.3
bio15	Precipitation seasonality (coefficient of variation)	3.7
bio4	Temperature seasonality (standard deviation *100)	3.4
bio8	Mean temperature of wettest quarter	2
bio16	Precipitation of wettest quarter	0.3

**FIGURE 4:**
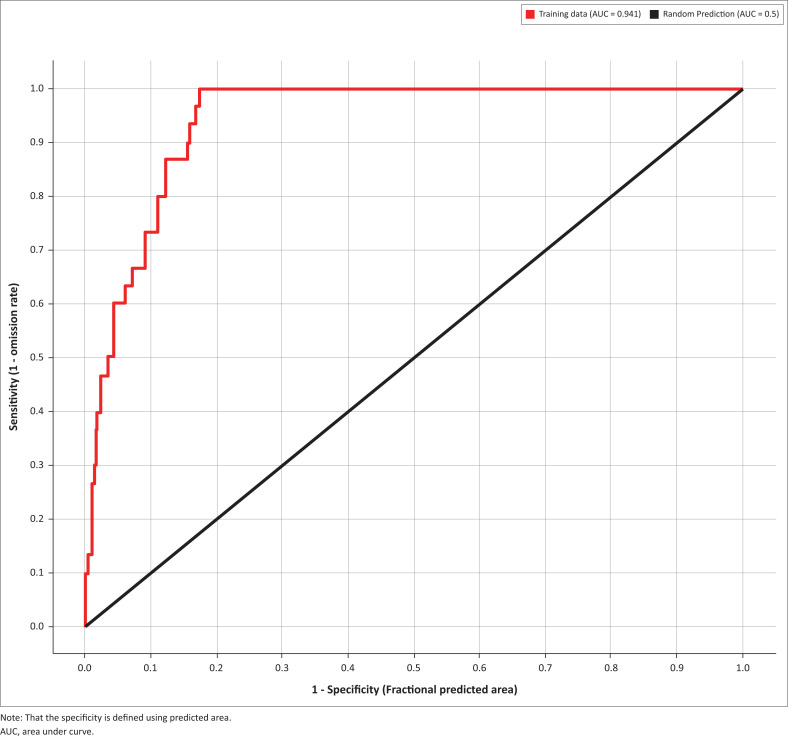
The receiver operating characteristic of the predicted model.

The MaxEnt model for *C. imicola* probability distribution in Tunisia provided satisfactory results, with an AUC value of 0.941 (±0.001), which is higher than 0.5 of a random model (Figure 4). Precipitation of Warmest Quarter (Bio18) contributed most to the model, followed by the Mean Temperature of Warmest Quarter (Bio10) and the Annual Precipitation (Bio12). The cumulative contribution of these three factors is 86% (Table 3).

**FIGURE 5:**
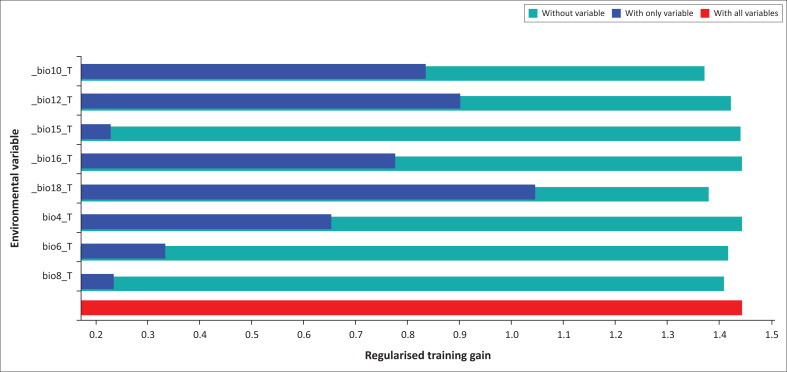
The Jackknife test for evaluating the relative importance of environmental variables for *C. imicola* distribution in Tunisia (The regularised training gain describes show much better the MaxEnt distribution fits the presence data compared with a uniform distribution. The dark blue bars indicate that the gain from using each variable in isolation. The light blue bars indicate the gain lost by removing the single variable from the full model, and the red bar indicates the gain using all of the variables).

The results of the jackknife test of variables’ contribution are shown in Figure 5. Bio18 provided very high gains (> 1.0) when used independently, indicating that this bioclimatic variable contained more useful information in itself than the other variables did. Bio10 and Bio12 had moderate gain when used independently (0.8 > gain < 1). Other variables including Bio16 and Bio4 had low gains when used in isolation; they did not contain much information by themselves.

### Current suitable areas for *Culicoides imicola*

**FIGURE 6:**
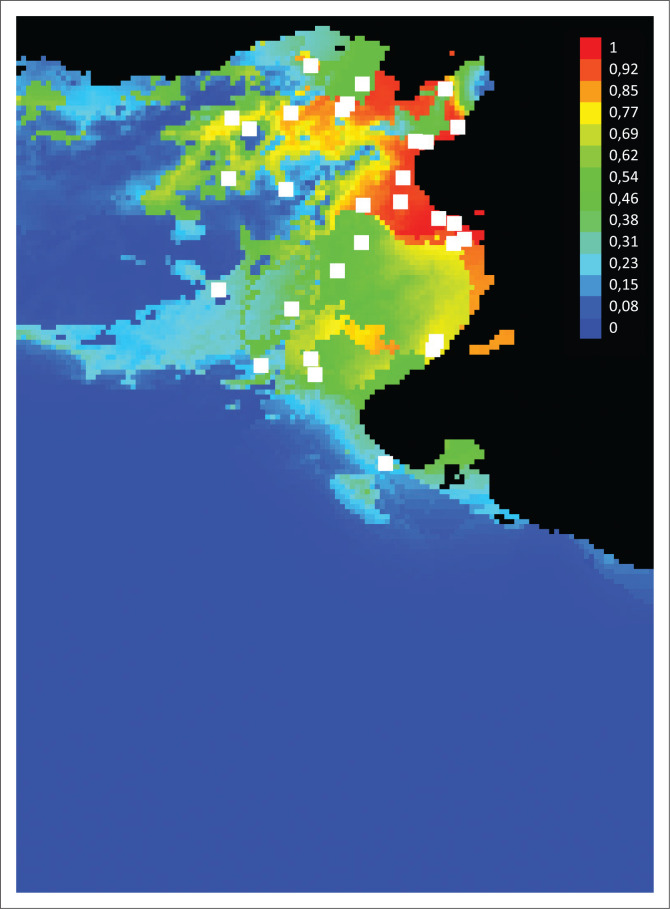
Maximum Entropy model for *C. imicola* probability suitable area presence in Tunisia (White dots show the presence locations used for training).

Based on the major environmental variables that modulate distribution of *C. imicola*, suitable habitats for this species were predicted in Tunisia. This is a representation of the MaxEnt model for *C. imicola*. Warmer colours show areas that have better predicted conditions (Figure 6).

## Discussion

Studies of the regional climatic niche of *C. imicola* have been conducted to determine its distribution in many countries using correlative modelling methods (Guichard et al. 2014). However, our study is the first to model the climatic niche of *C. imicola*, based on its general distribution in Tunisia. We employed two complementary model analyses: ENFA and MADIFA, to explore the relationship between the ecological niche of *C. imicola* and the availability of habitats (Calenge & Basille 2008). The ENFA and the MADIFA statistical tools use PO data (Basille et al. 2008; Hirzel et al. 2002). The ENFA and MADIFA can be suitable and efficient with data for which absences are unreliable or unknown (Hirzel et al. 2002). Furthermore, it is not necessary to know the limiting factors of the species but just to have an idea of which parameters could influence the species establishment and maintenance.

Our results suggest that the ecological niche of *C. imicola* is very different from the available habitat. The most important predictors driving the distribution of *C. imicola* in Tunisia were related to the Precipitation of Warmest Quarter (Bio18) (61%), followed by Mean Temperature of Warmest Quarter (Bio10) and the Annual Precipitation (Bio12). The cumulative contribution of these three factors is 86%. During the warmer months (June–September), the precipitation was the most influential factor determining the distribution of *C. imicola* in Tunisia. Field observations indicate that the population of *C. imicola* peaks in the September–October period and it seems that the summer rainfall has a direct impact on this population. This is in accordance with the observations from Sghaier et al. (2017) who noted that *C. imicola* peaks in Tunisia in October and that the maximum of BT cases in sheep in Tunisia are recorded in October and November (OIE 2018). Precipitation may influence distribution through an impact on the availability of breeding sites. Ducheyne et al. (2013) demonstrated that precipitation, especially summer rain-fall (June–September) was the most influential factor determining *C. imicola* distribution in Spain. This is in agreement with the observations made by Calvete et al. (2008, 2009) who noted that the coefficient of variation and the total amount of precipitation significantly influenced the presence of *C. imicola* (Calvete et al. 2008, 2009). In relation to precipitation values, more complex considerations should be made. In fact, although precipitation values may be used as predictors for species’ presence. *Culicoides imicola* populations peaked at the end of the rainy warm season (September–October). The fact that mean rainfall was negatively associated with *C. imicola* abundances could confirm that rainfall can inhibit the activity of some *Culicoides imicola* species as shown by Murray (1991).

The second important predictor driving the distribution of *C. imicola* in Tunisia is related to the Mean Temperature of Warmest Quarter (Bio10). This variable is negatively correlated to species occurrence. High temperatures combined with elevated dryness (typical from certain areas of Central and south of Tunisia) are fatal for *Culicoides* species. In general, temperature is known to influence greatly the development and survival of all stages of the life cycle of *Culicoides* species (Verhoef, Venter & Weldon 2014), driving the dynamics and distribution of many *Culicoides* species (Paweska, Venter & Mellor 2002). Temperature not only influences flight activity but also significantly affects the development time of immature stages, vector competence and adult survival. Modelling attempts often rely on a mean temperature threshold of 12.5 °C to predict presence or absence of *C. imicola* (Peters et al. 2011; Wittmann, Mellor & Baylis 2001) as established by Purse et al. (2007).

Statistical analysis of our data suggested that *C. imicola* was significantly more abundant in coastal areas at lowers altitudes. The same observations were reported in Sardinia where *C. imicola* and *Culicoides newsteadi* occur more frequently in coastal areas, whereas *Culicoides obsoletus* and *Culicoides pulicaris* are restricted to more mountainous central areas (Ramilo et al. 2012). The coastal Mediterranean zone exhibited the coolest maximum temperatures during summer. The low probability of occurrence observed in central and south regions in Tunisia is probably because of the very dry climate and hot temperatures registered in this region, especially in summer. The presence of permanently arable land with water sources nearby is favourable for this species development in the summer. However, high temperatures combined with elevated dryness (typical from certain areas of central and south of Tunisia) are fatal for *Culicoides* species.

It is important to keep in mind that there might be other variables that cannot be captured by satellite imagery and that may have an influence on the occurrence of these species on a local scale, such as soil conditions (affecting breeding sites) and farming practices. It is assumed that crop irrigation practice is a supporting factor for *C. imicola* presence. High-risk areas are shown in the governorate of Sidi Bouzid, where most of BT cases have been reported in Tunisia (OIE 2018). The effect of crop irrigation practice as a risk factor of *C. imicola* presence in Tunisia should be further investigated. *Culicoides imicola* shows some preference for different trees and, again for drier environments, probably used for breeding. This species breeds in areas where sunny surfaces prevail together with low vegetation (Ippoliti et al. 2013). *Culicoides imicola* avoid areas covered by forest complex (Conte et al. 2007; Meiswinkel, Venter & Nevill 2004). The negative influence of some permanent crops (olive groves) in *C. imicola* occurrence shows that this species may have preferences when choosing the best vegetation for breeding, oviposition and for larval and pupae development, which must be further evaluated.

The high abundance of *C. imicola* is most likely linked to its preference for breeding in areas where soil is moist and nutrient-rich and with full exposure to sunlight, characteristics, which are more commonly found in the centre and coastal areas of Tunisia. This hypothesis should be further investigated.

## Conclusion

We present here the potential ecological niche for *C. imicola* in Tunisia. The map presented here can be used to determine areas where the vector can be found. This would facilitate the implementation of control and surveillance programmes by Tunisian veterinary authorities. It is important to note that the high abundance of *C. imicola* in some regions of Tunisia underlines the real risk of spreading a new disease. Thus, it is important to improve our understanding of climatic factors in *C. imicola* activity influencing its distribution and seasonal pattern. Further studies are needed to continue the monitoring of other potential vectors in an attempt to limit the potential incursion and spread of the disease in other regions of Tunisia. We expect this approach to be promising in predicting the potential distribution of other arthropod vectors and can be an effective tool to assess and visualise the risk of the establishment and spread of VBD.
